# Hydrogen embrittlement and strain rate sensitivity of electrodeposited copper: part II – hydrogen dynamics

**DOI:** 10.1038/s41529-026-00799-4

**Published:** 2026-05-02

**Authors:** Desmond D. C. Williams, Ali Riahi, Anatolie Carcea, Taylor Martino, Nicholas A. Senior, Jason D. Giallonardo, Peter Keech, Suraj Y. Persaud, Mark R. Daymond, Roger C. Newman

**Affiliations:** 1 Department of Chemical Engineering and Applied Chemistry, University of Toronto, Toronto, ON Canada; 2 Natural Resources Canada, CanmetMATERIALS, Hamilton, ON Canada; 3 Nuclear Waste Management Organization, Toronto, ON Canada; 4 Department of Mechanical and Materials Engineering, Queen’s University, Kingston, ON Canada

**Keywords:** Chemistry, Energy science and technology, Materials science

## Abstract

In part I of this study, a previously unexplained form of hydrogen embrittlement and strain rate sensitivity was observed in electrodeposited copper. Here, the authors explored the dynamics of retained hydrogen in electrodeposited copper. Thermal desorption analyses were performed to characterize hydrogen trap energies of 0.39 ± 0.05, 0.63 ± 0.04, and 1.1 ± 0.04 eV. Annealing was conducted to evaluate the possibility of baking out hydrogen from electrodeposited copper and restoring ductility. A new electrodeposited material which is a candidate for coating used nuclear fuel containers was synthesized using tailored electrodeposition parameters, resulting in only 3.03 ± 0.58 ppm hydrogen, as compared to 26.4 ± 1.0 ppm. The mobility of hydrogen as it relates to embrittlement is discussed, and it is proposed that only hydrogen stored in low-energy trap sites contributes to embrittlement.

## Introduction

In Part I of this work^1^, a previously unexplained form of hydrogen embrittlement and strain rate sensitivity was observed in electrodeposited (ED) copper that was fabricated during a study on the manufacturability of high-purity copper on steel. Copper coatings such as these are candidate materials to be used in the disposal of nuclear waste^2–8^ thus, there is considerable interest in understanding manufacturing tolerances (e.g., surface preparation, bath chemistry/pH, current loading, etc.) and the consequences of producing samples in conditions that are out-of-specification. Part I of this work demonstrated that under certain process conditions, ED-Cu can incorporate abnormally high levels of hydrogen in its deposited microstructure, particularly as a result of the pH of the deposition electrolyte, and when present at high enough concentration this retained hydrogen can cause embrittlement. Slow strain rate tensile (SSRT) tests proved that the severity of hydrogen embrittlement in ED-Cu is dependent on both the strain rate and the temperature at which the test is conducted, such that higher temperatures and lower strain rates result in lower strain to failure (tests were conducted from 25 °C to 200 °C, and 5 × 10^−^^7^ to 5 × 10^−4 ^s^−1^).

The mechanisms by which hydrogen embrittles materials have been the focus of much research^9–14^ and while this work does not attempt to ascribe any one mechanism of hydrogen embrittlement to ED-Cu, evaluating the trapping and mobility of retained hydrogen can provide valuable insights into the capacity for hydrogen to embrittle metals. Such analysis will be pertinent across several hydrogen embrittlement mechanisms.

Broadly speaking, in many metals the embrittling capacity of hydrogen is most severe at near-ambient temperatures. Gangloff and Wei found that crack growth rates of 18Ni(250) maraging steel exposed to hydrogen pressures ranging from 12 to 133 kPa were fastest at near-ambient temperature^15^. For pipeline steels, Xing et al. found that X70 steel strained in 0.5 M H_2_SO_4_ and 0.1 g/L CH_4_N_2_S and hydrogen charged at 10 mA/cm^2^ exhibited the most severe embrittlement at 293 K, reaching 5% strain to failure as compared to the 8% strain to failure achieved at 313 K^16^. Similar observations have been made on other pipeline steels (which are routinely affected by hydrogen embrittlement) including X90 in simulated groundwater^17–19^. It is possible that at elevated temperatures the rate of hydrogen recombination on the metal surface is increased, resulting in a lower rate of hydrogen uptake and a lower retained hydrogen concentration within the metal. It is also possible that room temperature conditions provide the ideal thermodynamic environment to support hydrogen concentrating at crack tips, without adversely accelerating the rate at which hydrogen can detrap and diffuse away.

Body-centred cubic (BCC) materials are generally more sensitive to hydrogen embrittlement when compared to face-centred cubic (FCC) materials in part due to the much higher diffusivity of hydrogen within the BCC lattice^20^. These differences are attributed to the positioning of interstitial hydrogen atoms within the unit cell: Hydrogen occupies a tetrahedral interstitial site in BCC metals, and an octahedral interstitial site in FCC metals^21^. Since there is a larger activation energy barrier to hydrogen diffusion between adjacent octahedral interstitial sites in the FCC structure as compared to the tetrahedral interstitial sites in the BCC structure, the diffusivity of hydrogen within FCC metals is comparatively lower than BCC metals^22^. Interestingly, hydrogen, which occupies an octahedral interstitial position in the FCC copper^23^, does not migrate directly between adjacent octahedral interstitial sites in copper. Density functional theory studies of copper suggest instead that hydrogen migrates through an intermediate tetrahedral interstitial site, as this pathway offers a lower activation energy to migration^24,25^.

While the diffusion of hydrogen in a perfect crystal lattice is simple to understand, in practice, the mobility of hydrogen within conventional materials is far more nuanced. It is true that hydrogen can migrate between interstitial positions as suggested in the above discussion, but hydrogen can also become trapped at microstructural features, thereby limiting its effective diffusivity. Darken and Smith showed that hydrogen diffusion could be impeded by lattice imperfections, resulting in lowered diffusivity^26^. Building on this, McNabb and Foster proposed a continuum model for diffusion of hydrogen in solids incorporating the effect of hydrogen trapping on diffusivity^27^. In effect, hydrogen becomes trapped at these locations (e.g., grain boundaries, vacancies, dislocations, etc.) and does not de-trap until sufficient internal energy is supplied to the hydrogen atom, or some other microstructural re-arrangement takes place such as grain growth (for elevated temperatures) or slip^28^. If trapped hydrogen has sufficient energy to migrate to an interstitial lattice position, it can develop a local equilibrium between the trap site and the surrounding lattice. Oriani recognized the significance of traps on the diffusion of hydrogen within steels, and proposed a model for effective diffusivity as a function of mobile hydrogen as well as trapped hydrogen, wherein the two are in local equilibrium^29^.

Hydrogen trapping can be studied using thermal desorption spectroscopy (TDS). Kissinger was the first to derive an equation for the activation energy of a reaction analyzed by differential thermal analysis, and the same equation can be applied to TDS^30,31^. Kissinger’s mathematical derivation hinges on the observation that the temperature of maximum desorption rate within a desorption spectrum changes with heating rate, and in effect thermal desorption spectra can be used to evaluate the activation energy of individual temperature-dependent reactions^32,33^. Computational modeling of Kissinger-type thermal desorption experiments is in excellent agreement to the McNabb-Foster model for both diffusion-controlled and detrapping-controlled hydrogen mobility^34^. When the products of thermal desorption are characterized by non-spectroscopic methods, the term thermal desorption analysis (TDA) may be applied. TDS applying the Kissinger method has been performed extensively on metal-hydrogen systems. Hultquist et al. applied TDS to copper which had been exposed to humid air of 30–50% relative humidity for 10 years, which only retained 0.63 ppm hydrogen, and which is quite low as compared to instances of hydrogen embrittlement^35^.

There are very few studies on hydrogen contained within electrodeposited copper, and while there might be some similarities in trap energies between ED-Cu materials, the variation in microstructure with differing deposition parameters will result in unique desorption characteristics for any given ED-Cu material, as this will change the hydrogen trap distribution. Perhaps most importantly for this work, the effect of temperature on internal hydrogen embrittlement of ED-Cu has not been addressed in the literature to date. Among very few relevant studies, Fukai et al. did perform TDS on an electrodeposited copper formed in a solution of 0.9 M CuSO_4_ and 0.6 M H_2_SO_4_ with 120 ppm Cl^-^ ions at 313 K and a current density of 200 mA/cm^2^ and compared this to a commercially available copper thermally charged with hydrogen at 5 GPa and 900 °C for 2 hours. From their results they suggested that electrodeposited materials, which contain high concentrations of vacancies as well as voids, would trap greater concentrations of hydrogen at these sites than conventional cast copper. While this is a sensible conclusion based on direct observation, it is hasty to characterize specific desorption peaks as detrapping from vacancies or vacancy clusters without direct evidence. This oversimplified treatment would also neglect the other differences between as-cast and electrodeposited materials including differences in grain size and grain character^36,37^.

This work intended to evaluate the internal dynamics of hydrogen within ED-Cu: that is, the kinetics of hydrogen diffusion, trapping, detrapping, and desorption. Thermal desorption spectroscopy was conducted on ED-Cu to identify microstructural traps and characterize hydrogen trap energies. Possible internal hydrogen reactions with retained oxygen are discussed. Slow strain rate tensile testing (SSRT) was conducted on electrodeposited copper synthesized using tailored deposition parameters. Annealing heat treatments were conducted to study the reversibility of hydrogen embrittlement, and probe for possible high-temperature reactions of impurities within ED-Cu. Hydrogen dynamics are discussed with reference to hydrogen embrittlement.

### Results and discussion

#### Chemical analysis

The hydrogen and oxygen concentrations of the two ED-Cu materials studied in this work were measured using a LECO analyzer and are displayed in Table 1. Despite being synthesized in identical electrolytes, TDP-Cu retained only 3.03 ± 0.58 ppm hydrogen, compared with the 26.4 ± 1.0 ppm retained within Ac-Cu, proving that tailoring the electrodeposition parameters can lower the concentration of retained hydrogen. Similarly, only 7.53 ± 0.84 ppm oxygen was retained in TDP-Cu, compared to the 117 ± 3.3 ppm retained within Ac-Cu. It is possible for hydrogen to recombine with oxygen present in internal copper-oxide to form steam; however this reaction did not occur for samples studied here. The composition of Ac-Cu was presented in part I of this work^1^, and is repeated here. ICP-OES results displayed in Table 2 show that the elemental compositions of Ac-Cu and TDP-Cu are similar for the range of detected elements. Notably, Ac-Cu contained a higher nominal concentration of most detected impurity species, except for zinc, which was twice as concentrated in TDP-Cu. No secondary phases which would constitute unique hydrogen trap sites were observed in TDP-Cu by SEM. However, it is still possible that substitutional alloying elements could influence the trapping character of hydrogen.

**Table 1:** Elemental concentrations of oxygen and hydrogen retained within ED-Cu, detected using a LECO analyzer and tabulated in mass ppm

Material	H	O
Ac-Cu	26.4 ± 1.0	117 ± 3.3
TDP-Cu	3.03 ± 0.58	7.53 ± 0.84

**Table 2:** Elemental concentrations detected by ICP-OES, tabulated in mass ppm

Material	Ag	Al	Fe	Mg	Ni	P	Pb	S	Sb	Se	Si	Zn
Ac-Cu	< 1	5.2	28.3	27.6	44.9	< 1	5.2	3.5	46.1	8.7	38.2	29
TDP-Cu	3.6	3.3	19.5	15.4	30.5	< 1	1.6	2	33.4	3.6	34.1	64.6

#### Thermal desorption analysis

Experimental data prove that samples with a high concentration of retained hydrogen are embrittled but it is unclear by what mechanism hydrogen participated in embrittlement, and in what capacity hydrogen is stored in Ac-Cu. TDA was conducted on Ac-Cu to characterize trap sites of retained hydrogen, in an effort to better understand the nature of internal hydrogen dynamics. The theory supporting differential thermal analyses such as TDA were first defined by Kissinger, and culminated in Eq. 1^31^–an arithmetic derivation of Kissinger’s method is included in the supplementary information section.

1 $${\mathrm{ln}}\left(\frac{\varphi }{{T}_{m}^{2}}\right)={\mathrm{ln}}\left(\frac{AR}{E}\right)-\frac{E}{R{T}_{m}}$$

**Fig. 1:**
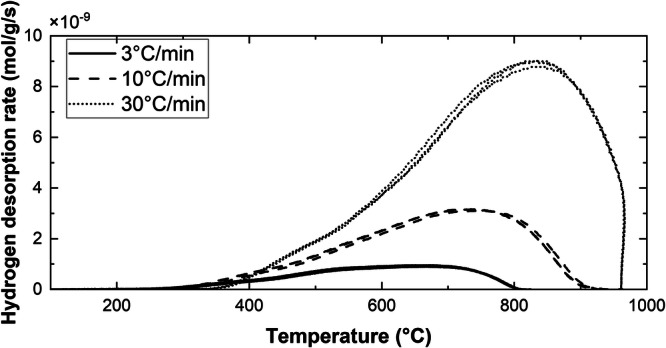
TDA conducted on Ac-Cu at varying ramp rates. The experiments are displayed here in triplicate, demonstrating reproducibility.

Where $\varphi$ is the ramp rate in K/s, ${T}_{m}$ is the temperature at the maximum desorption rate for a given peak in the desorption spectrum, $A$ is a constant, $R$ is the ideal gas constant, and $E$ is the activation energy of the trap site. The usefulness of this equation is evident when the $\mathrm{ln}\left(\frac{\varphi }{{T}_{m}^{2}}\right)$ is plotted against $\mathrm{ln}\left(\frac{1}{{T}_{m}}\right)$, such that the slope of the trendline equates to $-\frac{E}{R}$, and the activation energy of a given reaction may be quantified. TDA was conducted at 3 ramp rates between 3, 10, and 30°C/min, the results of which are found in Fig. 1. Each desorption experiment was repeated in triplicate.

**Fig. 2:**
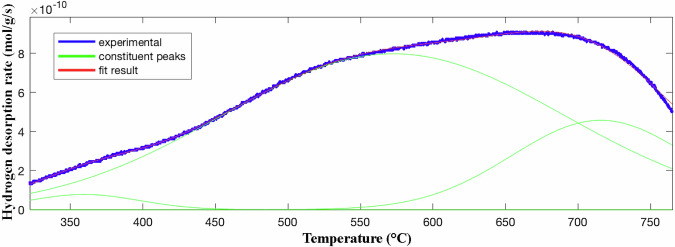
An example of Gaussian peak fitting to 3 °C/min TDA data. The fit achieved a coefficient of determination of R^2^ = 0.997.

**Fig. 3:**
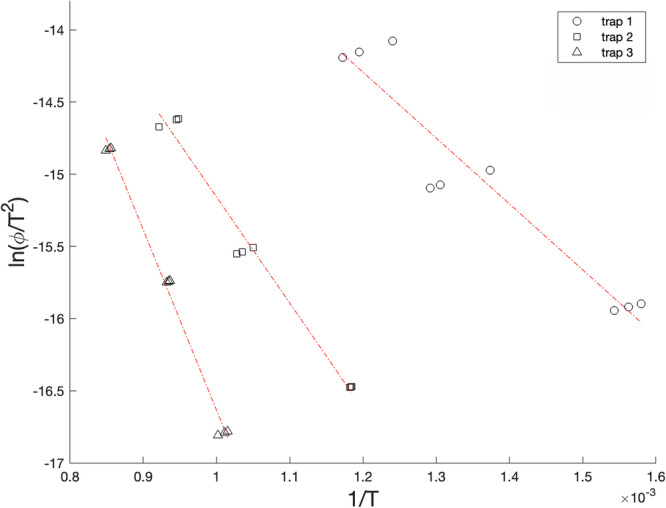
Kissinger plot giving activation energies from TDA data. The slope of the trendline fitted to each dataset is proportional to the activation energy of the trap site.

The TDA curves displayed in Fig. 1 were analyzed using a Gaussian peak fitting program in Matlab^38^. The Kissinger method assumes that the desorption peaks associated with individual trap sites are Gaussian, which is not necessarily the case practically, and is a known limitation of the Kissinger method. Using a trial-and-error approach, it was determined that 3 trap sites were present between 25° and 1000 °C, as this produced the most accurate fit of the desorption curve. An example result of the curve fitting is shown in Fig. 2. The Gaussians are centered about a peak temperature for each trap site, which corresponds to T_m_ in the Kissinger equation. While the peak detrapping temperature for a given trap site corresponds to the mean trapping energy for that trap class, the broadening of the Gaussian could be a result of the energetic distribution within that trap class. For example, not all grain boundaries possess the same trap energy; this will depend on grain character. Similarly, vacancy clusters of different sizes may possess different trap energies within the trap class. Plotting the results from Fig. 1 by Kissinger’s method yields Fig. 3. The slope of the trendline which fits the desorption data was calculated using the ordinary least squares (OLS) method, and the standard error of the slope of the trendline was determined. Presuming a Kissinger correlation, activation energies of trapped hydrogen were calculated and are shown in Table 3.

**Table 3:** Hydrogen trap energies in Ac-Cu

Trap Site	Trap energy (eV)
1	0.39 ± 0.05
2	0.63 ± 0.04
3	1.1 ± 0.04

The Kissinger method of TDA does make a major assumption, in that the outward diffusion of detrapped hydrogen is not considered. This assumption is made on the basis that the rate of lattice diffusion of hydrogen is higher than the rate of detrapping, and therefore the kinetics of detrapping can be simplified to a single rate-determining step. For example, if one considers the trap energy of 0.63 ± 0.04 eV given in Table 3 compared with the activation energy of hydrogen diffusion in copper given by Katz at 0.40 eV, then the rate of hydrogen diffusion would be 86 times higher than that of detrapping at 600 °C^39^. For the higher energy trap 1.1 ± 0.04 eV, the rate of detrapping is more than 5 orders of magnitude higher than that of lattice diffusion at 600 °C. From this method, higher energy traps can be characterized with greater certainty, and lower energy traps are more speculative due to the weakening of the primary assumption of Kissinger’s method.

The microstructural feature characteristic to each trap energy noted in Table 3 is not discernible from these data alone and must be coupled with experiments or atomistic simulations to verify trap locations. However, the lowest energy trap calculated as 0.39 ± 0.05 eV is similar to that of the lattice diffusion of copper at 0.40 eV^39,40^. It is possible that this trap could be representative of interstitial hydrogen. DSC experiments conducted by Benchabane et al. showed that the activation energy for recrystallization of cold-rolled copper was 0.60 eV, indicating that the 0.63 ± 0.04 eV may be representative of grain growth which frees hydrogen trapped at grain boundaries.

#### Tailored electrodeposition parameters

**Fig. 4:**
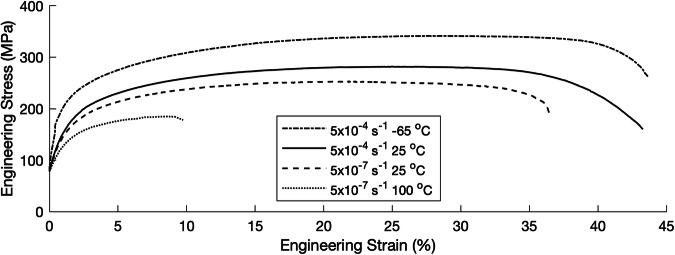
SSRT tests conducted on TDP-Cu at varying temperatures and strain rates. Reduced strain to failure is observed when tested at 100 °C and 5 × 10^−7 ^s^−1^.

**Fig. 5:**
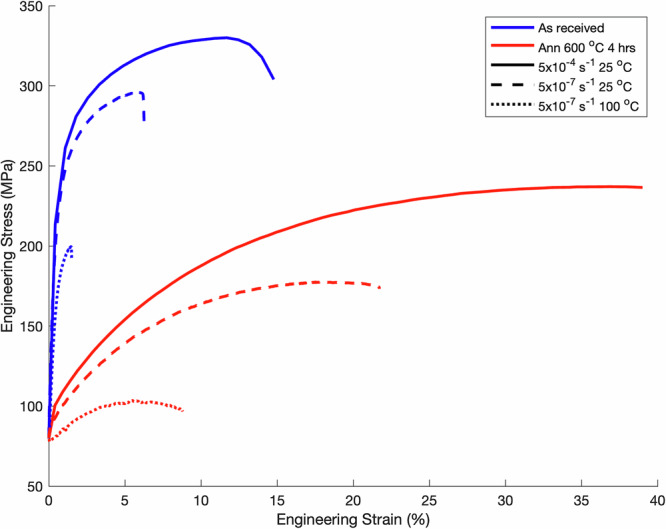
SSRT of Ac-Cu annealed at 600 °C for 4 hours. As-received Ac-Cu is also shown for comparison.

The tailored electrodeposition parameters used to synthesize TDP-Cu resulted in a retained hydrogen concentration of only 3.03 ± 0.58 ppm, which is nearly an order of magnitude lower than the 26.4 ± 1.0 ppm in Ac-Cu. SSRT tests were conducted on TDP-Cu and can be viewed in Fig. 4. Notably, TDP-Cu is relatively ductile at room temperature and 5 × 10^−^^4 ^s^−1^, reaching 43.7% elongation to failure - a significant improvement from Ac-Cu which reached only 15% elongation to failure for the equivalent test (Fig. 5). When tested in dry ice, the strain to failure of TDP-Cu is unchanged (Fig. 4), indicating that this level of retained hydrogen does not cause embrittlement at room temperature. There is some strain rate sensitivity observed in TDP-Cu when tested at 5 × 10^−7^ s^−1^, where strain to failure is reduced by 7%, but not as significant as was observed for Ac-Cu. There is however a loss of ductility observed when TDP-Cu is tested at 100 °C and 5 × 10^−7^ s^−1^, where it reaches only 10% strain to failure (Fig. 4).

**Fig. 6:**
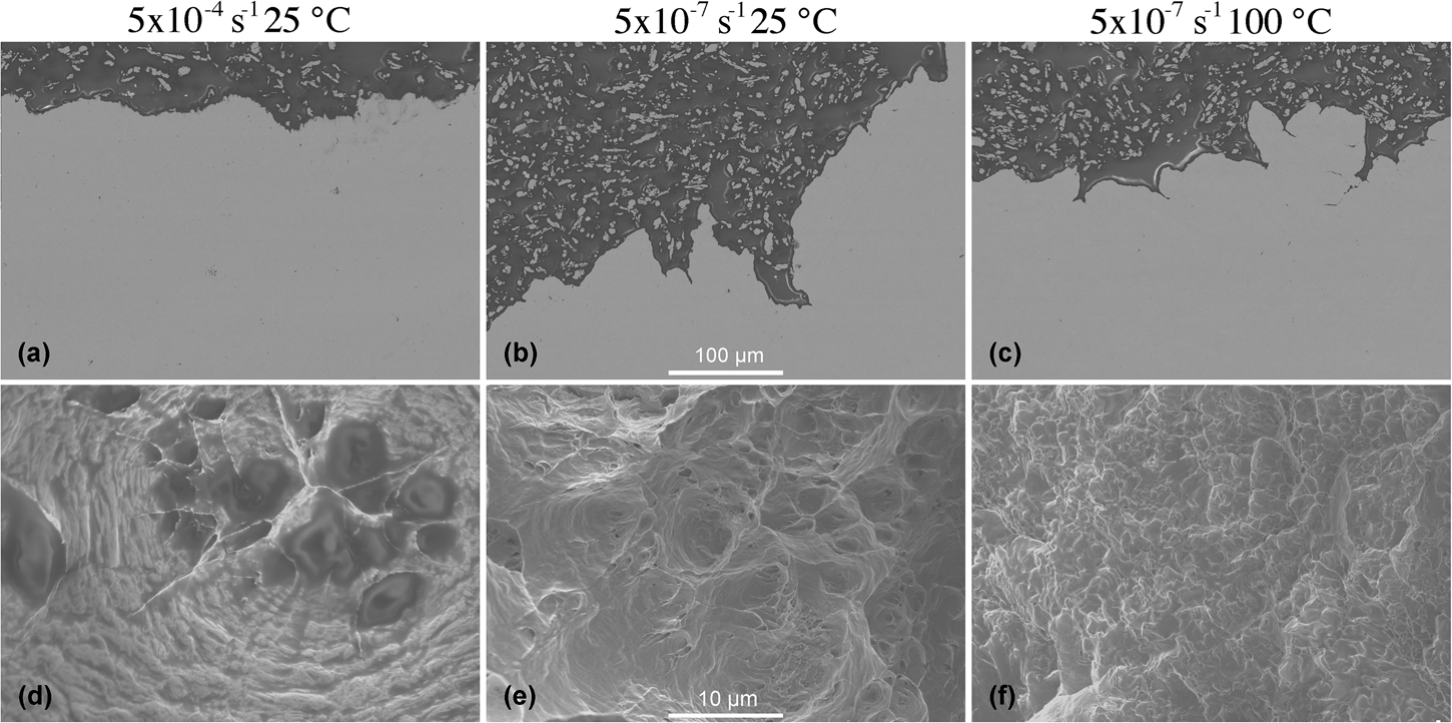
SEM images of TDP-Cu SSRT samples. (**a**–**c**) show cross sectional images, and (**d**–**f**) show fracture surfaces, where the test conditions are shown at the top of each column.

SEM imaging of TDP-Cu SSRT samples can be viewed in Fig. 6. When tested at 5 × 10^−4^ s^−1^ and 25 °C, the fracture surface is heavily dimpled, representative of ductile fracture, and the cross-sectional image shows no preference for grain boundary cracking. At 5 × 10^−7^ s^−1^ for both 25 °C and 100 °C, the crack path becomes increasingly tortuous, and while this may suggest the crack propagates intergranularly, this conclusion is difficult to discern from fractography alone.

#### Annealing of ED-Cu

**Fig. 7:**
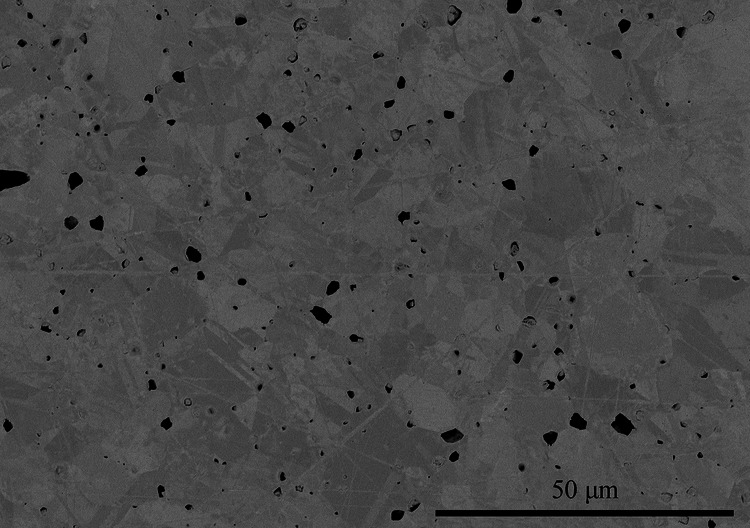
SEM micrograph of Ac-Cu annealed at 600 °C for 4 hours. Internal cavities were nucleated during annealing.

In an effort to overcome embrittling effects, Ac-Cu was annealed at 600 °C for 4 hours in a nitrogen atmosphere to desorb trapped hydrogen. Annealed Ac-Cu was then analyzed by LECO, and showed a retained hydrogen concentration of 7.49 ± 1.3 ppm. It was considered that annealing at 600 °C would have necessarily resulted in some microstructural evolution, including grain growth, which could alter the mechanical properties of the material. Other changes to microstructure could also occur including dislocation recovery and stress relaxation, which could in turn change the deformation character of the material. Cross-sectional SEM of annealed Ac-Cu is shown in Fig. 7. Many internal microvoids were nucleated during the annealing procedure, though these cavities are not spherical as would be expected for pressurised cavities.

It is well known that retained hydrogen can recombine with oxygen, or Cu-oxides, in copper metal to form steam cavities^41,42^. To investigate this possibility, LECO analysis was conducted on Ac-Cu before and after annealing at 600 °C for 4 h, and while 18.4 ppm hydrogen was desorbed during annealing, the concentration of retained oxygen was effectively unchanged – these data are listed in Table 4. Since the LECO analyzer removes moisture before the gaseous analyte approached the detector, any steam nucleated during annealing would bypass the detector and be missing from the post-anneal analysis. The unchanged oxygen concentration before and after annealing therefore, suggests that no hydrogen is converted to water, and the internal cavities in Fig. 7 are not a result of steam nucleation. Note that the hydrogen content of Ac-Cu prior to annealing reported in Table 4 differs from values reported elsewhere for this material. This discrepancy arises from the use of a different sample set. However, the range of hydrogen concentrations in both datasets falls within the standard error.

**Table 4:** Elemental compositions of hydrogen and oxygen in Ac-Cu before and after annealing at 600 °C

Material condition	Hydrogen	Oxygen
Before annealing	24.3 ± 2.3	82.8 ± 3.0
After annealing	7.49 ± 1.3	82.0 ± 3.1

Three samples were tested, and error is tabulated as statistical error, and mass is given as mass ppm.

There is the possibility that hydrogen could recombine to form gaseous hydrogen within nano-scale cavities, which are a product of electrodeposition. In this case, the pressure of the hydrogen gas within these cavities would necessarily have to exceed the yield stress of copper for the cavities to grow. If the yield stress of Ac-Cu at 600 °C is approximated as 120 MPa^43^, 26.4 ppm of hydrogen should then, by ideal gas law, correspond to a total volume of 7.98 × 10^−13 ^L/g of gaseous hydrogen in solid copper. This could not be accounted for by the 2% cavities by area observed in Fig. 7, suggesting that hydrogen recombination alone could not account for the observed cavity nucleation.

Other contributors to the evolution of these internal cavities could be coalescence of vacancies, or the thermal decomposition of polyethylene glycol. Many studies on the subject demonstrate that PEG can decompose into a myriad of organic molecules when heated in vacuum, which, in their gaseous state, could produce these pressurised internal cavities^44^.

The annealed Ac-Cu samples were subject to SSRT testing, the results of which are shown in Fig. 5. Evidently, annealing, and reducing the hydrogen concentration from 26.4 ± 1.0 to 7.49 ± 1.3 ppm hydrogen, restores ductility somewhat. However, even at this lower retained hydrogen concentration, strain rate and temperature sensitivity are still observed. Copious internal cavities formed after annealing will inherently affect the tensile response of this annealed material by acting as stress concentrators, though this effect is less pronounced in a ductile material like copper than for a high-strength alloy^45^. The significant reduction in yield stress observed for the annealed Ac-Cu samples is likely due to grain growth and dislocation recovery that would be expected in Cu under this heat treatment^46^. Although these microstructural alterations modify the deformation behavior of the material, the hydrogen-induced strain rate sensitivity persists.

**Fig. 8:**
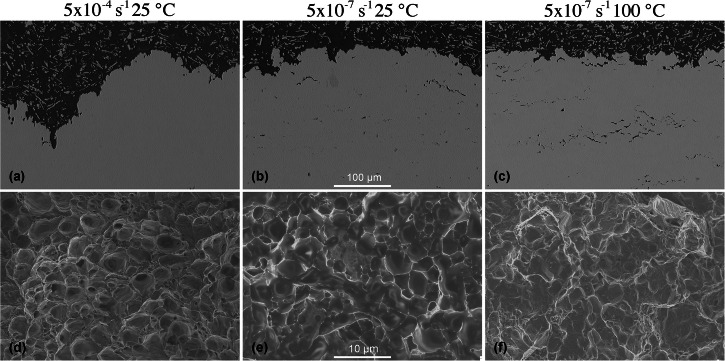
SEM imaging of Ac-Cu annealed at 600 °C for 4 h. (**a**–**c**) show cross sectional images, and (**d**–**f**) show fracture surfaces, where the test conditions are shown at the top of each column.

The apparent role of the internal cavities is further evident from the SEM images shown in Fig. 8; the prevalence of internal microcracks for tests conducted at 5 × 10^−^^7^ s^−1^ suggest that the cavities act as strain concentrators which nucleate cracks internally. More internal microcracks are observed for annealed Ac-Cu tested at 100 °C and 5 × 10^−7^ s^−1^ than at 25 °C, possibly due to the greater capacity of hydrogen to participate in deformation when it is more mobile around crack tips. While all fracture surfaces displayed in Fig. 8 are typical of ductile fracture, there is a preference for intergranular fracture at slow strain rate and high temperature. This is probably because internal microcracks form at grain boundaries, which contain elevated concentrations of hydrogen since they act as hydrogen traps. The greater embrittling capacity of hydrogen at this test condition then results in a greater fraction of intergranular fracture.

#### The role of mobile hydrogen

**Fig. 9:**
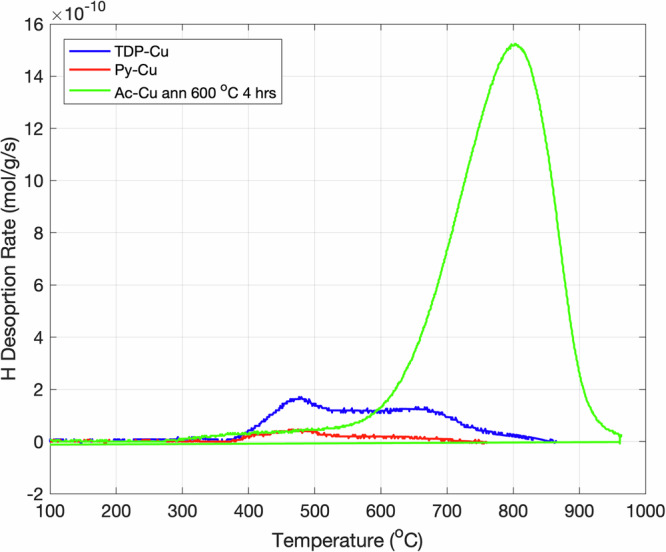
TDA spectra for TDP-Cu, Py-Cu, and Ac-Cu annealed at 600 °C for 4 h. These materials contained 3.03 ± 0.58, 5.25 ± 0.97, and 7.49 ± 1.3 ppm total bulk hydrogen respectively. Thermal ramping was conducted at a rate of 10 °C/min.

At only 3.03 ± 0.58 ppm hydrogen, one might not expect to see any embrittlement of TDP-Cu. However, one must consider the quantity of mobile hydrogen to determine the degree of embrittlement. Figure 9 shows the TDA spectra for TDP-Cu, Py-Cu – a material which contained 5.25 ± 0.97 ppm hydrogen and was the subject of a previous study^1^ – and Ac-Cu annealed at 600 °C for 4 h which contained 7.49 ± 1.3 ppm hydrogen. Py-Cu exhibited no signs of hydrogen embrittlement, while TDP-Cu exhibited some strain rate sensitivity at high temperature. This could be explained by the fact that TDP-Cu contained more low-energy trapped hydrogen than Py-Cu, which is evidenced by the TDA spectra shown in Fig. 9. Between 100°–1000 °C, 4.53 × 10^−8^ mol H/g was detected from Py-Cu, while 2.60 × 10^−7^ mol H/g was detected from TDP-Cu. Roughly six times as much hydrogen was desorbed from low-energy trap sites in TDP-Cu than Py-Cu for the equivalent desorption experiment, indicating that more hydrogen is stored at low-energy trap sites in TDP-Cu than Py-Cu, despite the fact that Py-Cu contained a higher nominal concentration of hydrogen. This suggests that only mobile hydrogen contributes to embrittlement, and that hydrogen stored in low-energy trap sites — being most readily mobilized under the test conditions of this study — is therefore most responsible for embrittlement overall. This finding is further supported by the observation that annealed Ac-Cu demonstrated the most significant strain rate sensitivity and embrittlement of materials shown in Fig. 9; 1.68 × 10^−6 ^mol H/g was detected by TDA between 100° and 1000 °C.

The main findings of this work are as follows:

- Three hydrogen trap sites in Ac-Cu were characterized by TDA, with activation energies of 0.39 ± 0.05, 0.63 ± 0.04, and 1.1 ± 0.04 eV.
- Tailored electrodeposition parameters resulted in only 3.03 ± 0.58 ppm retained hydrogen. TDP-Cu reached > 10% strain to failure at all tested temperatures and strain rates but still exhibited some temperature and strain rate sensitivity.
- Annealing of Ac-Cu at 600 °C can release some hydrogen, but even at 7.49 ± 1.3 ppm hydrogen, post annealing, embrittlement is still observed for this sample.
- Annealing of Ac-Cu results in the formation of internal cavities, which could not be attributed to hydrogen bubbles alone. It is hypothesized that these are in part formed due to the thermal decomposition of PEG.
- TDP-Cu containing 3.03 ± 0.58 ppm hydrogen exhibited hydrogen embrittlement, while Py-Cu containing 5.25 ± 0.97 ppm hydrogen did not^1^. It is proposed that the greater concentration of hydrogen in low-energy traps in TDP-Cu enabled embrittlement, as only mobile hydrogen is theorized to affect tensile properties for tests conducted in this study.

### Methods

#### Materials

**Fig. 10:**
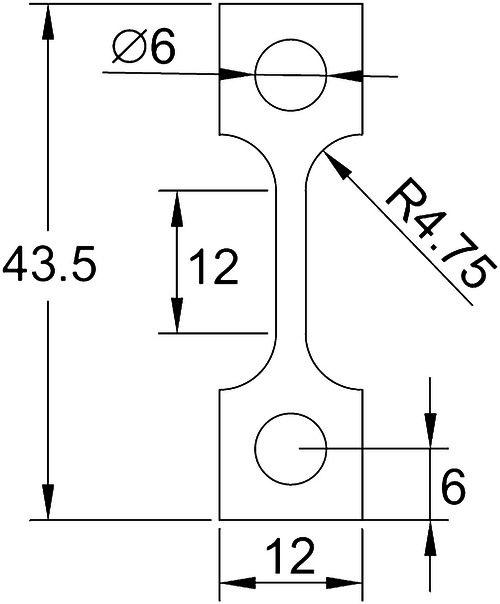
Dog bone SSRT sample used in this study (in mm). The thickness of the sample is 2 mm.

Two ED-Cu materials provided by the Nuclear Waste Management Organization (NWMO) were studied: “Acid-copper” (Ac-Cu), which was electrodeposited in an acidic electrolyte containing copper sulfate, sulfuric acid, chloride, and polyethylene glycol (PEG), and was the subject of much testing in part I of this work^1^; and “TDP-Cu”, a newly synthesized ED-Cu produced in the identical electrolyte as Ac-Cu, only using tailored electrodeposition parameters (TDP) of current and temperature. Both materials were synthesized from a high-purity copper consumable anode material and deposited on a cylindrical carbon steel substrate. The electrodeposited layer was removed from the carbon steel substrate using wire electrical discharge machining, and machined into dog bone samples, the dimensions of which can be found in Fig. 10.

#### Slow Strain Rate Tensile Testing (SSRT)

SSRT experiments were conducted using a test rig acquired from Cortest Inc (Willoughby, OH), which could be fitted with an Inconel 625 autoclave for experiments conducted at elevated temperature. Test data were collected using Load Frame Control Software version 11.5.05 from Cortest Inc., and all tests were repeated a minimum of two times to validate results. A pre-load of 40 kg was applied to straighten the drive train of the SSRT apparatus before testing at engineering strain rates of 5 × 10^−4^ s^−1^ and 5 × 10^−7^ s^−1^. A heating collar and insulating jacket were used to control temperature for tests conducted at 100 °C, and the autoclave was filled with a silicone oil to evenly distribute heat and eliminate temperature gradients within the SSRT sample. Samples tested in silicone oil were sonically cleaned in trichloroethylene followed by ethanol for 5 min at room temperature to dissolve any oil before further analysis. For tests conducted at −65 °C the autoclave temperature was gradually lowered by filling the autoclave chamber with dry ice and securing the lid, leaving the dry ice to sublimate. This process was repeated 5 times to stabilize the internal autoclave temperature before testing.

#### Chemical analyses

Internal hydrogen and oxygen concentrations were measured using a LECO ONH836 elemental analyzer, which uses infrared spectroscopy to detect select elements at sub-ppm sensitivity. A Thermo Scientific iCAP Pro Inductively coupled plasma optical emission spectroscopy (ICP-OES) instrument was used to characterize the chemical composition of as-received materials which were acid digested in 5% HNO_3_.

#### High-Temperature Thermal Desorption Analysis (TDA)

**Fig. 11:**
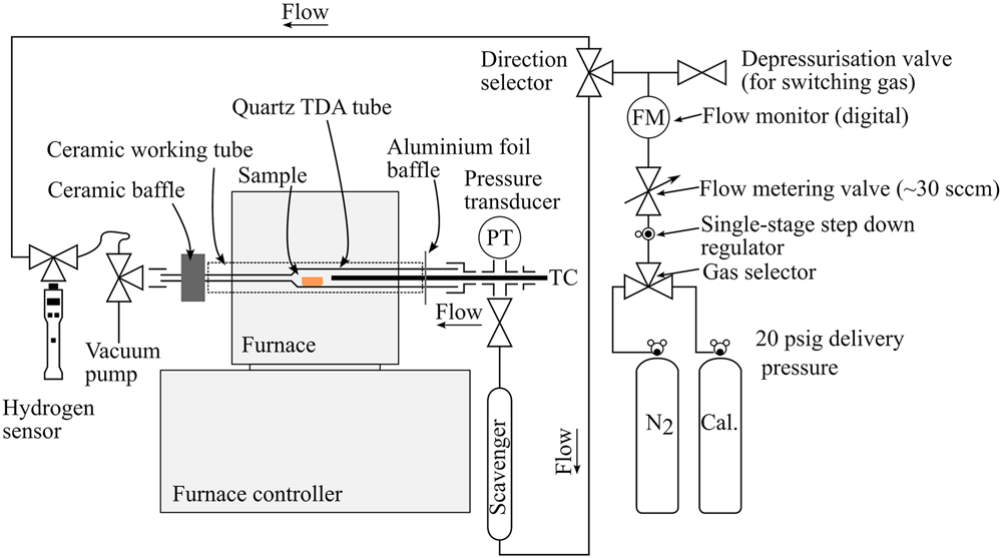
Schematic drawing of the TDA apparatus. The system consists of a once-through, open-ended flow line, wherein the carrier or calibration gas may be directed either through the quartz tube desorption chamber or bypassed directly to the hydrogen sensor.

A schematic drawing of the TDA apparatus is shown in Fig. 11. The tube furnace is a single-zone Carbolite MFT 10/15/130, with a built-in digital temperature controller. All piping (except for the TDA tube itself) is made of 316 L stainless steel and sealed with metal–metal surfaces. The TDA tube is constructed of quartz glass. The system is a once-through open-ended flow line. The system is first deoxygenated by pumping to vacuum within the TDA tube down to 0.09 bar (absolute), and re-pressurising with analytical carrier gas to atmospheric pressure. The carrier gas is 6.0 purity nitrogen (i.e., 99.9999%) and was passed through an oxygen/moisture scavenger immediately prior to entering the TDA tube. This vacuum and re-pressurizing procedure was repeated a minimum of four times, which reduces the initial oxygen concentration (of 20.9%) to single-digit ppm levels. After vacuum deoxygenation was completed, the nitrogen carrier gas was flowed through the tube at a volumetric flow rate of 30 ml/min.

The system is calibrated by flowing a calibration gas (nominally 100 ppm hydrogen in nitrogen) directly to the Hydrosteel® hydrogen probe, bypassing the furnace set-up entirely. This is done to calibrate the hydrogen sensor. This loop is then purged with nitrogen to fully remove any residual hydrogen from the piping before starting the TDA experiment. During analysis, the analytical carrier gas (ultra-pure nitrogen gas cylinder 99.9999%, Air Liquide) is flowed through the scavenger (for water and oxygen) and over the test specimen, carrying outgassed hydrogen to the Hydrosteel®. The detector is a Honeywell CiTiceL 4HYT electrochemical hydrogen probe. The output of the hydrogen sensor is recorded directly on the computer via an infra-red communication link. This additional module enables the Hydrosteel output to be recorded every 5 seconds indefinitely. Correlating experiments were carried out to validate the specimen temperature within the TDA apparatus with the furnace thermocouple, and ultimately, the furnace thermocouple was used to record specimen temperature.

The furnace was then energized, to heat the specimen at rates of 3, 10, or 30 °C/minute, to a maximum temperature (T_max_) of up to 1000 °C. The furnace was left at T_max_ until the recorded rate of hydrogen desorption had reduced to a constant baseline value (typically zero, or near zero if drift of the desorption rate was observed). The furnace was then de-energized and the sample left to cool, with continuously flowing carrier gas, typically until a temperature of 500 °C was attained, at which time the quartz glass tube was sealed. Once at room temperature (or < 50 °C), the tube was opened to air and the test specimen extracted and inspected for evidence of oxide. Any surface discoloration invalidated the test.

#### Scanning Electron Microscopy (SEM)

A Hitachi SU7000 SEM was used for imaging at accelerating voltages of between 5 and 10 kV and an aperture size of 50 μm. Samples which were to be imaged in cross-section were first mounted in a conductive epoxy then ground sequentially using Si-C paper of grit size 320, 600, and 1200. Polishing was conducted using diamond suspensions of 9 μm, 3 μm, and 1 μm, followed by a final surface treatment using colloidal silica of grit size 0.06 μm. Ultrasonic cleaning in ethanol was conducted between each polishing step to prevent carry-over of abrasives.

## Supplementary information

Supplementary Information.

## Data Availability

The datasets generated and/or analyzed during the current study are not publicly available because they are part of an ongoing research program, but are available from the corresponding author on reasonable request.
