# Supplementary material for: Hydrogen embrittlement and strain rate sensitivity of electrodeposited copper: part II – hydrogen dynamics
Source: Npj Mater Degrad. 2026 May 2;10(1):87. doi: 10.1038/s41529-026-00799-4 (PMC13341317; doi:10.1038/s41529-026-00799-4)
Supplement: Supplementary file 1 — Supplementary Information. [file 41529_2026_799_MOESM1_ESM.pdf]

Supplementary Information

Kissinger observed that the rate of thermal decomposition of a substance x is:

$$\frac{dx}{dt} = k(T)f(x) \tag{1}$$

Where $f(x)$ is the fraction of substance remaining, and *k(T)* is the temperature-dependent rate constant, given by the Arrhenius equation:

$$k(T) = Ae^{-\frac{E}{RT}} \tag{2}$$

Where E is the activation energy of the decomposition reaction, R is the ideal gas constant, T is the temperature and A is a pre-exponential frequency factor. Differentiating by parts yields the following equation:

$$\frac{d}{dt}\left(\frac{dx}{dt}\right) = Ae^{-\frac{E}{RT}}\frac{df(x)}{dt} + Af(x)\frac{d}{dt}\left(e^{-\frac{E}{RT}}\right) \tag{3}$$

$$\frac{d}{dt}\left(\frac{dx}{dt}\right) = \frac{df(x)}{dt} + f(x)\frac{E}{RT^2}\frac{dT}{dt} \tag{4}$$

Given that $f'(x) = \frac{f(x)}{dx}$ then $\frac{df(x)}{dt} = \frac{f' dx}{dt}$, The temperature ramp rate $\frac{dT}{dt}$ will be denoted φ. When the decomposition rate is at a maximum, its derivative with respect to time is zero. Evaluating the above equation at the peak desorption temperature T_m yields:

$$0 = Af(x)e^{\frac{-E}{RT}}f'(x) + x\frac{E\varphi}{RT_m^2} \tag{5}$$

Solving for $\frac{\varphi}{T^2}$ yields:

$$\ln\left(\frac{\varphi}{T_m^2}\right) = \ln\left(\frac{AR}{E}\right) + \ln(-f'(x)) - \frac{E}{RT_m} \tag{6}$$

For a first order reaction, $f(x) = (1 - x)$, and $f'(x)$ = -1, such that the above equation simplifies to the final form of the Kissinger equation:

$$\ln\left(\frac{\varphi}{T_m^2}\right) = \ln\left(\frac{AR}{E}\right) - \frac{E}{RT_m} \qquad (7)$$
